# BUILDING AN AGE-FRIENDLY ECOSYSTEM THROUGH NEIGHBORHOODS, HEALTH SYSTEMS, AND CAMPUSES

**DOI:** 10.1093/geroni/igad104.2611

**Published:** 2023-12-21

**Authors:** Valerie Greer, Terry Fulmer, Ashley Cadiz, Christopher Hernandez, Linda Edelman

**Affiliations:** University of Utah, Salt Lake City, Utah, United States; The John A. Hartford Foundation, New York City, New York, United States; University of Utah, Salt Lake City, Utah, United States; University of Utah, Salt Lake City, Utah, United States; University of Utah, Salt Lake City, Utah, United States

## Abstract

The age-friendly movement grew from an idea into a social movement recognizing the importance of adapting multiple facets of society to meet the needs of older adults. The movement integrates multidisciplinary work in research, policy, planning and design to build age-friendly ecosystems. Age-friendly ecosystems comprised of communities, health systems and campuses can become platforms for equity, health, and wellness for older adults. We designed a virtual symposium to examine age friendliness from a place-based approach by examining neighborhoods, campuses and health environments as sites uniquely positioned to catalyze equity in aging. The symposium was convened by national experts and regional aging stakeholders to consider how age friendly environments can address urgent social, environmental and health challenges, and create measurable outcomes that promote aging well for all. Knowledge was generated through lectures, panel discussions, a world café, focus group discussions, and a student-ideas competition. There were 113 attendees, including 27 health professions students; 78% of attendees indicated their work focused on aging/older adults and 52% reported specialized training in geriatrics/gerontology. Attendees reported increased familiarity with age-friendly concepts post-symposium. Key findings indicated the benefits of shared language and metrics across place-types that can collectively transform the social, physical, and economic landscape of aging. Symposium outcomes include a website, graphic facilitation, a collaboration resource guide, and a forthcoming book. Outcomes are serving as a foundation to radically re-imagine cross-sector collaborations between communities, health systems and campuses to create an ecosystem that embraces longevity as an asset and advances equity in aging.

